# Evaluation of Khat (Catha edulis) Use as a Risk Factor of Cancer: A Systematic Review

**DOI:** 10.31557/APJCP.2020.21.4.881

**Published:** 2020-04

**Authors:** Zhi Xiong Chong, Wan Yong Ho, Pan Yan, Mustafa Ahmed Alshagga

**Affiliations:** *Division of Biomedical Sciences, School of Pharmacy, University of Nottingham Malaysia. *

**Keywords:** Systematic review, Risk of Bias (RoB), OHAT, khat, premalignant condition, cancer

## Abstract

**Background:**

Conducting systematic review to evaluate plant use as a risk factor to cancer could be challenging. A systematic and well-balanced method should be applied to accommodate in vivo and in vitro studies to make a final decision. In this article, khat, a recreational plant used in some Arabic and African regions, was employed as an example to systematically determine its relationships to the premalignant and cancerous conditions.

**Methods:**

Systematic database search was performed to recruit original human, animal or in vitro studies on khat and cancer. Sixteen studies fulfilled the inclusion criteria and subjected to assessment using Risk of Bias (RoB). Office of Health and Translation (OHAT) approach was used to rate the confidence level in the body of evidence. The evidence was integrated to establish the relationships between khat, premalignant conditions and cancer.

**Results:**

Seven out of eight studies showed that khat causes premalignant oral lesions with moderate evidence level. Four studies showed that khat causes cancer with low evidence level and another three studies showed that khat has anti-cancer effect with moderate to high evidence level. Only one study suggested that khat is unrelated to cancer.

**Conclusion:**

RoB and OHAT approach are reliable systematic tools to evaluate plant risk to cancer and provide objective and uniform summary regardless of the study type. In conclusion, our pooled analysis did not find a direct relationship between khat and cancer but anti-cancer effect would require to be proofed on human studies.

## Introduction

Systematic review on herbal use and association to cancer are heterogeneous, and different study designs like in vitro and in vivo studies could be challenging as it is hard to draw an objective and fair conclusion without utilisation of an objective tool fits to evaluate different study designs and measurement of findings (Krauth et al., 2013; Viswanathan et al., 2013; OHAT, 2015a). In 2011, the Office of Health Assessment and Translation (OHAT), Department of Health and Human Services, United States, has introduced a systematic guidelines that use Risk of Bias (RoB) to objectively evaluate the relationship between the exposure of a substance and its relation to a disease (OHAT, 2015a, 2015b). 

Khat (Catha edulis) is a recreational natural plant that is being commonly consumed or chewed in some Arabic, Middle East and African regions (Al-Motarreb et al., 2002; Gebhard et al., 2010; Nakajima et al., 2016). Khat leaves have fragrance smell and sweet sensation that make palatable for people to consume, (El-Zaemey et al., 2015; El-Setouhy et al., 2016; Chong et al., 2017) and also it is producing a state of “euphoria” that encourages the users to crave for it (Al-Motarreb et al., 2002; Aziz et al., 2009; Ligani and Hussen, 2014; El-Setouhy et al., 2016) . 

**Figure 1:**
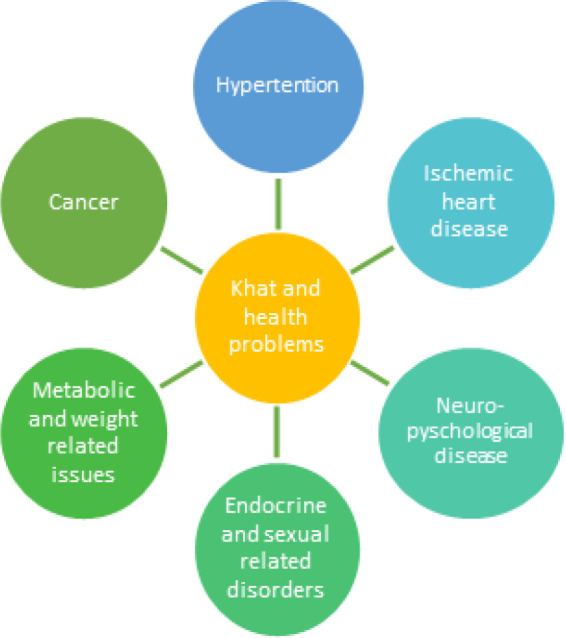
Health Disorders Associated with Khat Consumption (Al-Motarreb et al. 2002; Hassan et al. 2005; Getahun et al. 2010; Colzato et al. 2011; Hoffman and Absi 2011; Girma et al. 2015; Lukandu et al. 2015; Machoki et al. 2015; Alshagga et al. 2016).

Although khat research has gained a considerable attention during the past three decades, most of these studies were cause-effect in nature, and barely we could find mechanistic and molecular studies that provide more insight about risk/benefit of this plant. The spectrum of human diseases associated with khat use include (Figure 1):

Like many other natural products, khat contains many bioactive compounds like cathine, cathinone, norephedrine, pseudoephedrine, phenols, flavonoids, non-toxic metals, minerals and vitamins (Lukandu et al., 2008; Bredholt et al., 2009; Birhane and Birhane, 2014; El-Zaemey et al., 2015; Sallam et al., 2016; Chong et al., 2017; Al-Maweri et al., 2018). These substances especially the flavonoids and phenolic compounds are not only important for the plant to grow and survive in the environment, but also provide a number of useful health benefits to the users like antioxidant, anti-inflammatory, anti-microbial and anti-cancer properties (Elhag et al., 1999; Dimba et al., 2004; Choe and Min, 2009; Zhang et al., 2011; Cheng et al., 2013; Lu et al., 2017).

Since khat was reported to contain multiple bioactive compounds that exhibit anti-cancer properties, it creates a research question on how does khat is associated/predispose to cancer? This interesting and contradicting relationships is rarely explored in the reported literature. Therefore, to fill in the literature gap, this present systematic review was established to use a standard tool of assessment on all published studies with the aim of understanding the relationship between khat and premalignant conditions (hyperkeratosis, dysplasia, leukoplakia, oral white lesions and melanin depositions) or cancer.

Here, we are describing how to apply RoB and OHAT approach to integrate the various evidences about khat and premalignant conditions or cancer from human, animal and in vitro studies and considering variations in the different study designs (Rooney et al., 2014; OHAT, 2015a; Stephens et al., 2016). 

## Materials and Methods

*Study Protocol*

**Figure 2:**
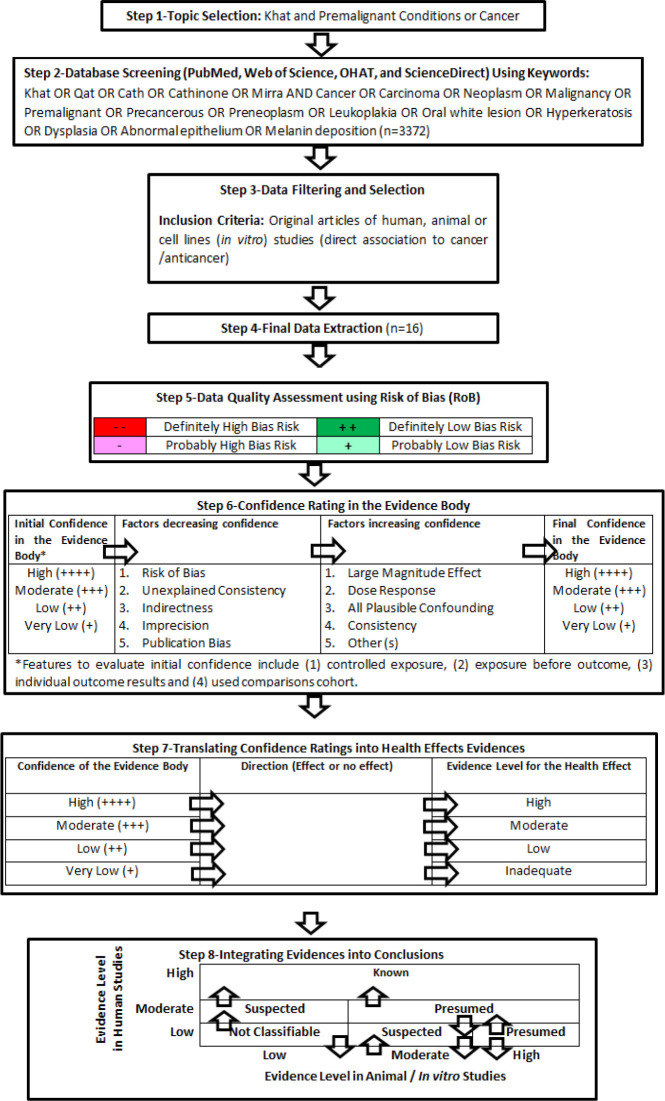
Approaches Employed to Study the Relationships between Khat and Cancer. The Steps were Constructed Based on the OHAT Approach in Systematic Review (OHAT 2015a, 2015b) and Two Recent Systematic Reviews which Employed the Similar Approach were Referred to Complement the Current Study Design (Vaccari et al., 2017; Wikoff et al., 2018).

A systematic literature search using RoB and OHAT approach (Figure 2) was performed to identify the potential relationships between khat and premalignant conditions or cancer.

*Database Search and Final Sources Selection*

Upon topic selection, four database search in which four different databases, namely, (1) PubMed, (2) Web of Science, (3) OHAT and (4) ScienceDirect, were used to look for the suitable association studies on khat and premalignant conditions or cancer. The keywords used in the search included “khat” OR “Qat” OR “Cath” OR “Cathinone” OR “Cathine” OR “Mirra” AND “Cancer” OR “Carcinoma” OR Neoplasm” OR “Malignancy” OR “Pre-malignant” OR “Pre-cancerous” OR Pre-neoplasm” OR “Leukoplakia” OR “White Oral Lesions” OR “Hyperkeratosis” OR “Dysplasia” OR “Abnormal Epithelium” OR “Melanin Deposition” OR any closely-related keywords. About 3400 articles were extracted, those studies reported only khat alone or cancer-related health problems, but not both keywords or no causation, therefore, were excluded. For this reason, around 3,000 articles were excluded. 300 studies that appeared in multiple database were considered as duplication and were excluded further. Out of the 100 articles left, literature review, case reports, expert’s opinion, short communications, meeting abstracts and articles which were not reported in English were excluded. Only original human, animal or in vitro (cell lines) research studies on khat and cancer were selected. In the final stage of the data selection, sixteen suitable studies were fulfilled the criteria and selected for the review. 

*Quality Assessment Using Risk of Bias (RoB)*

The sixteen selected studies underwent quality assessment using RoB as described by the guidelines (OHAT, 2015a, 2015b), in order to determine the internal validity of each selected study. The internal validity assessment was performed by two independent reviewers and any queries was resolved through a third reviewer to ensure consistencies across different reviewers. The questions of the quality assessment could be divided into six main domains and the question list can be found at: 

https://ntp.niehs.nih.gov/ntp/ohat/pubs/handbookjan2015_508.pdf 

Each question is rated using different symbols and colours: “definitely high RoB” (- -, dark red), “probably high RoB” (-, light red), “probably low RoB” (+, light green) and “definitely low RoB” (+ +, dark green). Based on the questions answered with the ratings given, each study is given a RoB tier: Tier I-most of the domains are answered as “probably low” or “definitely low” RoB and no domain is evaluated to be “definitely high” RoB, Tier II-a study that does not fit both Tier I and III criteria, and Tier III- most of the domains are answered as “probably high” or “definitely high” RoB. 

*Confidence Rating in the Evidence Body*

The sixteen selected khat studies were grouped based on the premalignant condition or cancer types, namely (1) premalignant oral lesions, (2) oro-pharyngeal cancer, (3) oesophageal cancer and (4) leukemia and breast cancer. For the initial confidence rating, four features were taken into consideration (OHAT, 2015a, 2015b; Wikoff et al., 2018) and these included: (1) controlled exposure, (2) exposure before outcome, (3) individual outcome results and (4) use of any comparison group (s). The initial confidence is rated as “high, ++++” if it includes all the four features, “moderate, +++” if it includes three features, “low, ++” if it includes any of the two features and “very low, +” if it only covers one feature. 

After the establishment of the initial confidence rating, different factors were used to either increase or decrease the confidence rating. Factors that decrease confidence include (1) risk of bias, (2) unexplained consistency, (3) indirectness, (4) imprecision and (5) publication bias. Studies with high RoB, unclear study direction, unexplained consistencies and imprecisions are more likely to downgrade the confidence rating in the evidence body. Factors that increase confidence include (1) large magnitude of effect, (2) dose response, (3) all plausible confounding, (4) consistency and (5) other. This means if the gathered evidences from various studies show high magnitude effect of a substance to a health state with clear and consistent dose-dependent effects, the confidence rating in the evidence rating is more likely to be upgraded.

Based on the adjustment in the confidence rating, each evidence body is given a final confidence rating as either “high confidence, ++++”, “moderate confidence, +++”, “low confidence, ++” or “very low confidence, +”. 

*Integration of Confidence Rating and Conclusion Drawing*

The confidence rating was used to rate the level of evidence for health effect in determining whether khat has effect or no effect on different cancer types (Figure 2). The evidence was then integrated to draw a conclusion on whether khat has “non-classifiable”, “suspected”, “presumed” or “known” effect on cancer based on the different selected studies.

## Results

*Risk of Bias (RoB) Assessment*

From the sixteen selected studies, eight studies were about khat and premalignant oral lesions, three studies were focusing on khat and oro-pharyngeal cancer, two studies were about khat and oesophageal cancer, two studies were focusing on khat and leukemia and one study was about khat and breast cancer. Based on the Risk of Bias (RoB) assessment (Figure 3A and 3B), ten studies were classified as Tier II study and another six studies were classified as Tier III study. No study was grouped to be the Tier I study, which could be interpreted as no high quality study had approved the relationship between the plant and cancer.

For all the selected studies, there was no controlled clinical trial study, hence, the included studies protocols were not blinded, and this presumes to possible performance and detection bias during the study, for example, during the data collection. Besides, most studies were either case-control studies or cross-sectional studies or in vitro cell lines studies with little or inadequate randomisation, and this caused the studies to have high probable selection bias. In some case-control studies, for examples, in study by Machoki et al., (2015) and study by Leon et al., (2017), there was no proper matching of cases and controls for exposure to carcinogenic substances such as smoking among controls and khat users, thus, some of these studies were rated to have high probable performance bias. 

A common limitation of most collected human studies was the unreliable statistical analysis which not computing the risk by modelling the data finding to various time-exposure or various time-confounders in the study, diversity of chemicals and staging of disease (Wang et al., 2016).

*Relationships between khat and premalignant oral lesions*

Based on Table 1, it was shown that five out of the eight selected studies were assigned with moderate initial confidence ratings and Tier II RoB while the remaining three studies were given low initial confidence ratings and Tier III RoB. After checking with the points that could increase or decrease the confidence ratings, it was finally shown that khat has moderate confidence ratings in the body of evidence in causing the premalignant oral lesions. 

In the first listed study by Macigo et al., (1995) (n=226), khat was said to be not significantly associated (P>0.05) with oral leukoplakia compared to cigarette smoking (RR=8.4; 95% Cl=4.1, 17.4). This was the only one study out of the eight studies which demonstrated that khat consumption was not significantly related to the occurrence of the premalignant oral lesions. Subsequently, two case control studies by Gorsky et al., (2004) (n=102) and Scheifele et al., (2007) (n=200) showed that the incidence of premaglinant oral lesions was significantly higher (p<0.05) among chewers compared to non-chewers and smoking did not play a significant role in inducing the premaglinant oral lesions. The studies also suggested that khat induced premalignant oral lesions in dose- and time-dependent manner. 

**Table 1:** Confidence Rating in the Evidence Body of the Studies that Reported the Relationships between Khat Usage and Premaglinant Oral Lesions (n=8)

Study Type (Sample Size, n)		Author, Year	Finding (s)			Initial Confidence Rating a		Factors Decreasing Confidence b										Factors Increasing Confidence C										Final Confidence in the Body of Evidence
								Risk of Bias Tier		Unexplained Inconsistency		Indirectness		Imprecision		Publication Bias		Large Magnitude Effect		Dose Response		All Plausible Confounding		Consistency Across Study Types		Other(s)		
Case-control (n=226; case=85 and control=141)		(Macigo et al. 1995)	Khat was not significantly associated (p>0.05) with oral leukoplakia compared to cigarette smoking (RR=8.4; 95% Cl=4.1,17.4).			Low (++)		III		Only the study by Macigo et al. showed that khat usage did not significantly cause premalignant oral lesions		All studies reported the direct association between khat usage and premalignant oral lesions		Imprecision was not obvious as most studies used appropriate measurement tools and statistical analysis		The RoB ranged from Tier II to III		Magnitude effect of khat on the occurrence of the pre-		Khat usage was said to cause pre-		Confounding variables were considered, and control was employed in all studies		Results were quite consistent in supporting that khat usage was linked to pre-malignant oral lesions, except the study by Macigo et al. 1995		These 3 studies were case-control studies		Moderate (+++)
Case-control (n=102; case=47 and control=55)		(Gorsky et al. 2004)	Oral white lesions were significantly more evident (p<0.001) among khat chewers (83%) compared to non-chewers (16%). There was no significant association between the oral lesions and smoking.			Moderate (+++)		II										malignant lesions were large as most studies were human clinical studies		malignant								
Case-control (n=200; case=142 and control=58)		(Scheifele et al. 2007)	Prevalence of oral precancerous lesions increased significantly (p<0.05) with increased frequency and duration of khat consumption and was not associated with smoking.			Low (++)		III												oral lesions in dose dependent manner but the exposure dosage and duration were unclear								
Retrospective survey (n=300; 150 khat users and 150 non-khat users)		(Ahmed et al. 2010)	Incidence of oral white lesions, hyperkeratosis and cellular atypia were significantly higher (p<0.05) among the khat chewers than non-chewers.			Low (++) to Moderate (+++)		II		No obvious observed inconsistency and all 4 studies showed khat significantly increase risk of oral pre-		All studies directly determined the relationships between khat usage and premalignant oral lesions		No obvious Imprecision as most studies used appropriate measurement tools and statistical analysis		Not severe bias as most of the RoB was III		Khat usage was said to have large magnitude effect on the pre-malignant oral lesion occurrence but the direct pathogenesis was unclear		Khat usage was said to cause pre-		Different variables were considered, and control was employed in all studies		Results were consistent in supporting that khat usage was associated to pre-malignant oral lesions		Except the study by Ahmed et al. was a retrospec-tive study, the other three studies were cross-sectional studies		Moderate (+++)
Cross-sectional (n=650; 490 khat users and 160 non-khat users)		(Al-Sharabi 2011)	Almost all khat chewers (486 or 99.7%) had white patch on the buccal mucosa and this was significant (p<0.05) in relative to the non-chewers.			Low (++)		III		malignant lesions										malignant								
Cross-sectional (n=162; 109 khat users and 53 non-khat users)	(Schmidt-Westhausen et al. 2014)			White oral lesions were more significantly observed (p<0.001) among khat chewers (~80%) than non-chewers.	Moderate (+++)		II												oral lesions in dose- and time- dependent manner but the exposure duration and dosage were unclear in most studies									
Cross-sectional (n=42; 14 chronic khat users, 20 chronic khat users + smokers and 8 non-users)	(Lukandu et al. 2015)			Chronic khat chewing caused significant increase (p<0.05) in the oral mucosa epithelial thickness, hyperkeratinisation and melanin deposition.	Low (++) to Moderate (+++)		II																					
Cross-sectional study (n=1052; 547 khat user, 505 non khat user)	(Al-Maweri et al. 2017)			Presence of premalignant oral lesions are significantly associated with khat chewing only (p<0.001)	Low (++) to Moderate (+++)		II		No inconsistency as the study aim matched the study finding		The study directly determined the relationships between khat usage and premalignant oral lesions		No obvious Imprecision as the study employed appropriate measurement tools and statistical analysis		No severe bias as the study used appropriate study protocol and measurement method		The magnitude effect of khat on premalignant oral lesion was unclear as the dosage or exposure duration were unclear		The dose response relationship was unclear		Different variables were considered, and control was employed		Results were consistent in supporting that khat usage was associated to pre-malignant oral lesions		The study did consider smoking as another potential cause of premalignant oral lesion		Moderate (+++)	

^a^, Key features to rate initial confidence include (1) controlled exposure, (2) exposure prior to outcome, (3) individual outcome data and (4) comparison group used. The confidence level is “high, ++++” if it includes four features, “moderate, +++” if it includes three features, “low, ++” if it includes two features and “very low, +” if it only involves one feature; ^b^- Factors that decrease confidence include (1) risk of bias, (2) unexplained consistency, (3) indirectness, (4) imprecision and (5) publication bias; ^c^ - Factors that increase confidence include (1) large magnitude of effect, (2) dose response, (3) all plausible confounding, (4) consistency and (5) other.

**Table 2:** Summary of the Confidence Rating in the Body of Evidence for the Studies that Reported the Relationships between Khat and Oro-Pharyngeal Cancer (n=3)

Study Type (Sample Size, n)	Author, Year	Finding (s)	Initial Confidence Rating ^a^	Factors Decreasing Confidence ^b^					Factors Increasing Confidence ^C^					Final Confidence in the Body of Evidence
				Risk of Bias Tier	Unexplained Inconsistency	Indirectness	Imprecision	Publication Bias	Large Magnitude Effect	Dose Response	All Plausible Confounding	Consistency Across Study Types	Other(s)	
Oro-pharyngeal Cancer														
Retrospective survey (n=28)	(Soufi et al. 1991)	36% of non-smoking oro-pharyngeal cancer patients had history of khat chewing for at least 25 years.	Very Low (+)	III	Mild inconsistency could be caused by study design components	Poor study direction, some studies focused in evaluating the relationships between khat and genotoxicity, then only linked to cancer.	Precision could not be accurately measured as some study did not use statistical analysis.	Yes, as the RoB tier ranged from II to III	Magnitude effect was not large as some study was in vitro study that did not consider pharmaco kinetic factor.	Khat was said to cause cancer in dose- dependent manner.	The studies considered confounding factor and control was used in all study types.	Results are consistent in three study types, suggesting khat consumption could be related to oro-pharyngeal cancer	These three studies had different study studies, thus, it is hard to compare the study findings.	Low (++)
Cross-sectional (n=109)	(Kassie et al. 2001)	Khat consumption significantly increased (p=0.002) frequency of micronuclei (MN) in oral mucosa cells in dose-dependent manner, thus, increasing oral cancer risk.	Moderate (+++)	II										
*In Vitro*	(Lukandu et al. 2008)	Khat methanolic extract apoptosed (p<0.05) primary human oral keratinocytes (NOK) and fibroblasts (NOF) in concentration-depen dent manner, increased oxidat ive stress and this could lead to oral cancer.	Moderate (+++)	II										

^a ^– Key features to rate initial confidence include (1) controlled exposure, (2) exposure prior to outcome, (3) individual outcome data and (4) comparison group used. The confidence level is “high, ++++” if it includes four

features, “moderate, +++” if it includes three features, “low, ++” if it includes two features and “very low, +” if it only involves one feature; ^b^- Factors that decrease confidence include (1) risk of bias, (2) unexplained consistency, (3)

indirectness, (4) imprecision and (5) publication bias; ^c^ - Factors that increase confidence include (1) large magnitude of effect, (2) dose response, (3) all plausible confounding, (4) consistency and (5) other.

**Figure 3A:**
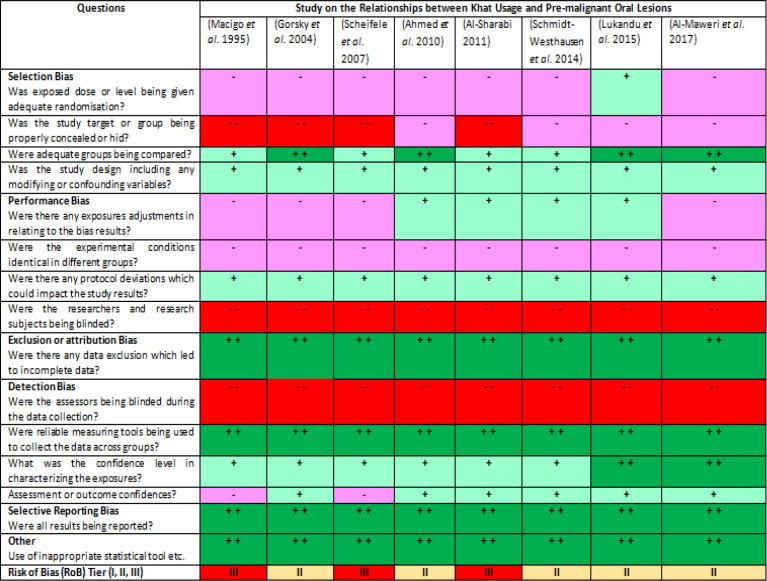
Heat Map of the Risk of Bias (RoB) of Different Studies that Reported the Relationships between Khat Usage and Premalignant Oral Lesions (n=8). All questions were adapted from the RoB guidelines (OHAT 2015b). Each question is assessed using different colour codes and symbols: “definitely high RoB” (dark red, - -), “probable high RoB” (light red, -), “probable low RoB” (light green, +) and “definitely low RoB” (dark green, + +). RoB tier is finalised and assigned based on the combined “scores” for example: Tier I – almost all questions are answered as “probably low” or “definitely low” RoB and no question is said to be “definitely high” RoB, Tier II-any study that falls between Tier I and III criteria, and Tier III- most of the questions are evaluated to have “probably high” or “definitely high” RoB

**Figure 3B:**
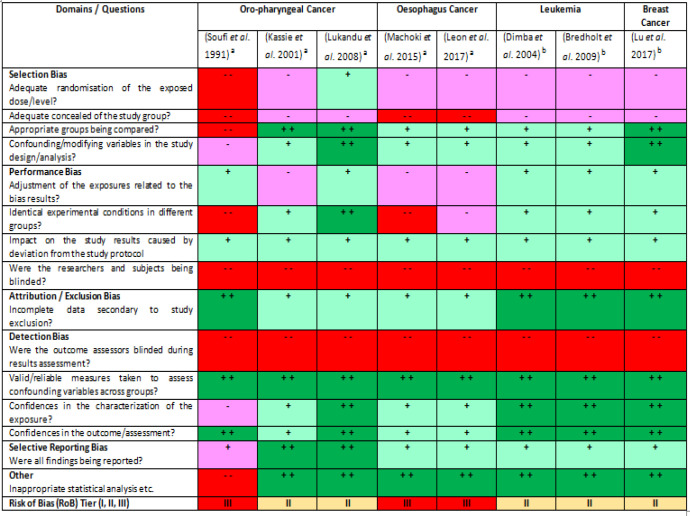
A Summary on the Risk of Bias (RoB) Heat Map of Different Studies that Reported the Pro-/anti-cancer Effects of Khat (n=8). ^a^-Khat pro-cancer; ^b^-Khat anti-cancer. The domains or questions were adapted from the RoB guidelines (OHAT 2015b). Each domain is evaluated using different symbols and colours: “definitely high RoB” (- -, dark red), “probably high RoB” (-, light red), “probably low RoB” (+, light green) and “definitely low RoB” (+ +, dark green). Based on the domains answered, each study is given a RoB tier: Tier I-most of the domains are answered as “probably low” or “definitely low” RoB and no domain is evaluated to be “definitely high” RoB, Tier II-a study that does not fit both Tier I and III criteria, and Tier III- most of the domains are answered as “probably high” or “definitely high” RoB

**Table 3:** Summary of the Confidence Rating in the Body of Evidence for the Studies that Reported the Relationships between Khat and Oesophageal Cancer (n=2)

Study Type (Sample Size, n)	Author, Year	Finding (s)	Initial Confidence Rating ^a^	Factors Decreasing Confidence ^b^					Factors Increasing Confidence ^C^					Final Confidence in the Body of Evidence
				Risk of Bias Tier	Unexplained Inconsistency	Indirectness	Imprecision	Publication Bias	Large Magnitude Effect	Dose Response	All Plausible Confounding	Consistency Across Study Types	Other(s)	
Oesophageal Cancer														
Case-control (Case=91; control=182)	(Machoki et al. 2015)	There was no significant association (p>0.05) between khat usage and oesophageal cancer.	Moderate (+++)	III	Not serious as both studies had almost similar study design components	Not serious, the studies directly evaluated relationships between khat and cancer	Yes, as 1 study did not find clear association between khat and cancer.	Quite severe, as the RoB tier were III for both studies.	Magnitude effect was unclear since both studies showed opposite findings	Khat was said to cause cancer in dose- dependent manner in one of the studies only.	The studies considered confounding factor and control was used in all study types.	1 of the studies reported no association between khat and cancer	Both studies were all case-control studies.	Low (++)
Case-control (Case=73; control=133)	(Leon et al. 2017)	Khat user had 2-fold higher risk to get oesophageal cancer compared to non-users (OR=2.12; 95% Cl=0.94, 4.74).	Moderate (+++)	III										

^a^ – Key features to rate initial confidence include (1) controlled exposure, (2) exposure prior to outcome, (3) individual outcome data and (4) comparison group used. The confidence level is “high, ++++” if it includes four features, “moderate, +++” if it includes three features, “low, ++” if it includes two features and “very low, +” if it only involves one feature; ^b^- Factors that decrease confidence include (1) risk of bias, (2) unexplained consistency, (3)

indirectness, (4) imprecision and (5) publication bias; ^c^ - Factors that increase confidence include (1) large magnitude of effect, (2) dose response, (3) all plausible confounding, (4) consistency and (5) other.

**Table 4:** Summary of the Confidence Rating in the Body of Evidence for the Studies that Reported the Relationships between Khat and Leukemia or Breast Cancer (n=3)

Study Type (Sample Size, n)	Author, Year	Finding (s)	Initial Confidence Rating ^a^	Factors Decreasing Confidence ^b^					Factors Increasing Confidence ^C^					Final Confidence in the Body of Evidence
				Risk of Bias Tier	Unexplained Inconsistency	Indirectness	Imprecision	Publication Bias	Large Magnitude Effect	Dose Response	All Plausible Confounding	Consistency Across Study Types	Other(s)	
Leukemia and Breast Cancer														
In vitro (Leukemia)	(Dimba et al. 2004)	Khat methanolic extract significantly apoptosed (p<0.05) human leukemic cell lines (HL-60, Jurkat and NB4 cells) in concentration- and time-dependent manner.	Moderate (+++) to high (++++)	II	Mild inconsistency could be caus ed by study design components.	Not serious, the studies directly evaluated relationships between khat and cancer.	No, all studies precisely showed that khat possesses anti-cancer effects on leukemic and breast cancer cell lines.	No severe as the RoB tier were II for all three studies.	Magnitude effect was prominent with increasing khat usage dosage and duration.	Khat inhibited leukemic or breast cancer cell lines at higher concentration.	The studies considered confounding factor and control was used in all study types.	All three studies reported khat killed leukemic or breast cancer cell lines.	All three studies were in vitro studies and used r eliable s tatistical analysis. However, no animal and human studies were reported on the anti-cancer effects of khat.	Moderate to high (++++)
In vitro (Leukemia)	(Bredholt et al. 2009)	Apoptosis was observed in khat methonolic extract-treated human acute myeloid leukemia cell lines (MOLM-13) significantly (p<0.05) in time-dependent manner.	Moderate (+++) to high (++++)	II										
In vitro (Breast cancer)	(Lu et al. 2017)	Khat ethanolic extract significantly induced mitochondrial-mediated apoptosis (p<0.01) in human breast cancer cell lines (MDA-MB-231) through activation of MAPK pathway.	Moderate (+++) to high (++++)	II										

^a^ – Key features to rate initial confidence include (1) controlled exposure, (2) exposure prior to outcome, (3) individual outcome data and (4) comparison group used. The confidence level is “high, ++++” if it includes four features, “moderate, +++” if it includes three features, “low, ++” if it includes two features and “very low, +” if it only involves one feature; ^b^- Factors that decrease confidence include (1) risk of bias, (2) unexplained consistency, (3)

indirectness, (4) imprecision and (5) publication bias; ^c^ - Factors that increase confidence include (1) large magnitude of effect, (2) dose response, (3) all plausible confounding, (4) consistency and (5) other.

**Figure 4:**
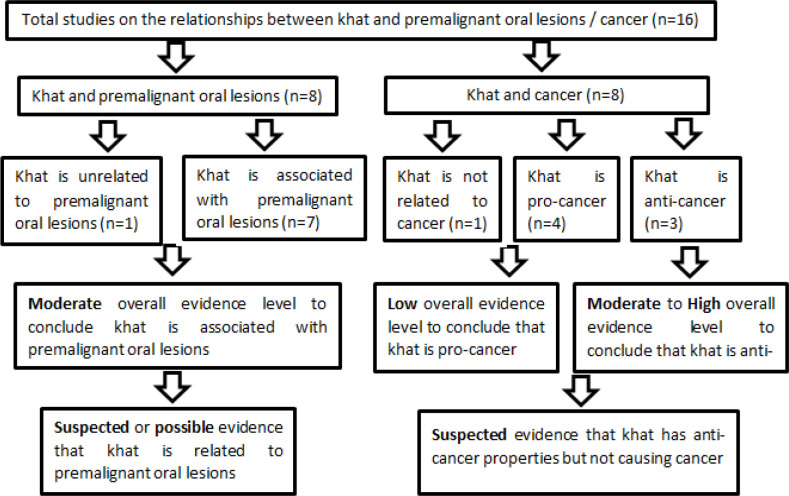
A summary of the Published Evidences on the Inter-relationships between Khat, Premalignant Oral Lesions and Cancer

## Discussion

Ahmed et al., (2010) reported a retrospective study with equivalent number of case and control (n=150 for each category) and they found that the incidence of oral white lesions, hyperkeratosis and cellular atypia were significantly higher (p<0.05) among the khat chewers than the non-chewers. However, it seems like the authors did not properly consider other factors like heterogeneous diseases, smoking when establishing the relationships between khat consumption and premalignant oral lesions. 

In the more recent years, four cross-sectional studies were reported that khat consumption would significantly increase the risk of premalignant oral lesions. In the first study by Al-Sharabi et al., (2011) (n=650), almost all khat chewers (486 or 99.7%) were found to have white patch on the buccal mucosa and these changes were significant (p<0.05) as compared to the non-chewers. The study also found that the oral lesions were unrelated to cigarette and water pipe smoking. In the study by Schmidt-Westhausen et al. in 2014 (n=162), premalignant oral white lesions were significantly observed (p<0.001) among khat chewers (about 80%) as compared to the non-chewers and the lesions mostly correlated to the side of chewing (75.2%) and chewing duration. This indirectly implies that khat-induced premalignant oral lesion is site-, dose- and time-dependent. Again, the authors reported that tobacco and water pipe smoking did not significantly influence the presence of the oral white lesions. In another cross-sectional study by Lukandu et al., (2015) (n=42), it was reported that chronic khat chewing significantly increased (p<0.05) oral mucosa epithelial thickness, caused hyperkeratinisation and melanin deposition. Like the previously reported studies, smoking did not significantly worsen the khat-induced oral histopathological changes. Al-Maweri et al., (2017) reported that among 547 khat users, the presence of premalignant oral lesion was significantly associated p<0.001) with khat chewing only (without smoking). 

Based on all the mentioned points, six out of the eight selected studies directly, consistently and precisely showed that khat caused premalignant oral lesions in dose- and time-dependent manner but the exact pathogenesis was unclear. Therefore, at this stage, it is said to have moderate confidence rating in the evidence body to support that khat is likely to induce premalignant oral lesions and the effect is said to be “suspected”. 

*Relationships between Khat and Oro-Pharyngeal Cancer*

Based on Table 2, three studies which reported the relationships between khat and oro-pharyngeal cancer were summarised. The three studies had different study designs in which one was a retrospective survey (Tier III RoB), one was a cross-sectional clinical sampling study (Tier II RoB) and the last one was an in vitro study (Tier II RoB). 

The retrospective study by Soufi et al., (1991) (n=28) reported that 36% of the non-smoking, oro-pharyngeal cancer patients, had history of khat chewing for at least 25 years. However, for other non-khat chewing, oro-pharyngeal cancer patients, the authors did not conclude on the possible reasons of the cancer like genetic or other environmental factors. Therefore, the relationships between khat use and oro-pharyngeal cancer was not strong based on this study finding. 

In the cross-sectional clinical sampling study by Kassie et al.,(2001) (n=109), khat consumption (100g/day) was found to significantly (p=0.002) increase the frequency of micronuclei (MN) and genetic damage in the oral mucosa cells of recruited human subjects in the dose-dependent manner, thus, increasing genotoxic effects and oral cancer risk among the users. The khat consumer group was matched with a control group for age, smoking and binge drinking histories, thus, minimize the possible variations between the two groups. 

In the in vitro study by Lukandu et al., (2008), khat methanolic extract (10-316µg/mL) significantly apoptosed (p<0.05) primary human oral keratinocytes (NOK) and fibroblasts (NOF) within 3-6 hours post-treatment and the effect was concentration- and time-dependent. The extract was reported to raise oxidative stress by generating reactive oxygen species (ROS) and depleting the glutathione (GSH) level which killed the normal oral mucosa cells. The mutagenic event then possibly induced carcinogenesis and caused oral cancer. Fibroblasts were said to be more sensitive to khat than keratinocytes and the apoptosis was not reversed by anti-oxidant. 

All three studies suggested that khat is related to oro-pharyngeal cancer with low to moderate initial confidence ratings. The direction of the three studies were not really clear as the relationships between khat and oro-pharyngeal cancer was not assessed directly in some study that focused on genotoxicity then only linked to oro-pharnygeal cancer. There might have some inconsistencies due to different study designs and imprecisions as the retrospective survey did not use statistical analysis. On the other hand, the three studies suggested that khat had dose- and time-dependent effects in causing genotoxicity and oro-pharyngeal cancer but the magnitude effect was not clear as one of the studies was just an in vitro study which did not consider the pharmacokinetic effect of khat in the human body. In overall, even though the three studies had different study designs, but all three studies suggested that khat use was associated with oro-pharnygeal cancer and the final confidence rating given for this body of evidence was low (++).

*Relationships between Khat and Oesophageal Cancer*

Association between khat and oesophageal cancer was reported by two case-control studies (Table 3) in which both studies were rated as Tier III RoB. In addition, both studies were given moderate initial confidence ratings.

In the case-control study reported by Machoki et al., (2015) (case=91; control 182), the association between khat usage and oesophageal cancer was reported to be insignificant (p>0.05). However, it was unreported on whether the control and khat-users group were matched for age, smoking and binge drinking histories, and this possibly raised bias issue on whether other genetic and environmental factors might influence the risk of getting oesophageal cancer. 

In another case-control study reported recently by Leon et al., (2017) (case=73; control=133), khat user was found to have two-fold higher risk to get oesophageal cancer compared to non-users (OR=2.12; 95% Cl=0.94, 4.74). Besides, the control and khat-users group were matched for age, smoking and binge drinking histories to increase the study validity and reliability. 

Generally, both case-control studies directly assessed the relationships between khat usage and oesophageal cancer, and khat was suggested to link to oesophageal cancer in a dose- and time-dependent manner with possibly magnitude effects on the human health in one of the studies. However, the p-value was not significant from the 95% confidence interval (Cl) and thus, the association between khat and oesophageal cancer was insignificant and the final confidence in the body of evidence was rated to be low (++).

*Relationships between Khat and Leukemia or Breast Cancer*

Based on Table 4, two studies were in vitro studies that described the anti-leukemic effects of khat methanolic extracts and another study described the anti-cancer effects of khat ethanolic extracts against breast cancer cell lines. All the three studies were rated as Tier II RoB and all studies were given moderate to high initial confidence ratings. 

In the first listed study which was reported by Dimba et al., (2004) the authors reported that khat methanolic extract (20µg/mL) could significantly trigger (p<0.05) apoptosis in human leukemic cell lines (HL-60, Jurkat and NB4 cells) within 8 hours post-exposure and the process was blocked by pan-caspases inhibitor (Z-VAD), caspase-1 inhibitor (Z-YVAD-fmk) and caspase-8 inhibitor (Z-IETD-fmk). The apoptosis was more sensitively triggered in leukemic cells than normal human peripheral leukocytes, and the effect was observed to be concentration- and time-dependent.

Few years later, in another study by Bredholt et al., (2009) apoptosis was observed in khat methonolic extract (200µg/mL)-treated human acute myeloid leukemia cell lines (MOLM-13) (p<0.05) and the process was mediated through c-FLIPL cleavage and procaspase-8 activation. Expressions of anti-apoptotic protein, induced myeloid leukemia cell differentiation protein (Mcl-1) was reduced by khat treatment.

Based on the findings described by the two studies, it was shown that instead of sustaining leukemic cells and causing more devastating effect, khat and its extract was found to have anti-leukemic effects on various cell lines and the effects were said to be dose-dependent. Since the study findings were quite consistent between the two studies, this suggested probable large magnitude effects of khat in killing leukemic cancer cells. 

In another study that involved khat extract and breast cancer cell lines by Lu et al., (2017), khat ethanolic extract (20-400µg/mL) significantly induced (p<0.05) mitochondrial-mediated apoptosis in human breast cancer cell lines (MDA-MB-231) through activation of MAPK pathway in concentration- and time-dependent manner. The extract increased expression of apoptosis key protein (Bax), extracellular regulated protein and activated c-Jun N-terminal kinases (JNK). Besides, khat extract decreased ROS level and expression of B-cell lymphoma 2 protein (Bcl2). This was the first study that reported the potential anti-cancer effects of khat extract on the breast cancer cell lines.

Since all the three studies directly assessed anti-cancer effects of khat and showed consistent trends that khat extracts have promising anti-cancer effects, the final confidence ratings of the three studies were rate to be high (++++). The only drawback is that these in vitro study findings would need to be supported by in vivo study in the future for results validation. 

In summary, based on the different studied evidences (n=16) (Figure 4), khat leaf itself or its extract were shown to cause premalignant oral lesions (n=7) and the overall evidence level to conclude this effect is rated to be moderate. Therefore, khat is suspected to have known evidence in causing premalignant oral lesions. However, out of the eight studies on khat and cancer, it is surprisingly to see that only 50% of the reported studies described that khat is related to different cancer like oro-pharyngeal and oesophageal cancer but the final evidence level is rated to be low. One study showed that khat is not significantly associated with oesophageal cancer but again, the final evidence level is low. On the other hand, three in vitro studies reported that khat has anti-cancer effect against leukemia and breast cancer and the evidence level for this is rated to be moderate to high. Considering the different evidences on the pro- and anti-cancer effect of khat and its extracts, it is therefore concluded that khat might have suspected anti-cancer effect. If khat has suspected effect in causing premalignant oral lesions and suspected anti-cancer effect, then, this will be a contradicting yet interesting topic to be discussed further. 

Consistent to present study finding, two previous studies (El-Zaemey et al., 2015; Al-Maweri et al., 2018) also suggested that khat is associated with premalignant oral lesions, except that the mentioned studies did not use OHAT approach in producing the systematic review. The possible mechanism on how khat predisposes to premalignant oral lesions is still unknown till today, but generally it could be caused by chemical or mechanical reasons (Al-Sharabi, 2011). For the chemical reason, it was proposed that tannin, one of the khat bioactive compounds, is able to induce oral mucosal epithelial changes, and subsequently drives carcinogenesis (Al-Sharabi, 2011; Al-Maweri et al., 2018). Besides, it has been reported that khat could increase oxidative stress and induce genotoxicity in the oral mucosa cells to drive neoplastic changes (Kassie et al., 2001; Lukandu et al., 2008). As for the mechanical reason, it was postulated that chronic khat chewing would produce frictional and abrasive injuries to the oral mucosa, and subsequently lead to epithelial hyperplastic and dysplastic changes (Al-Sharabi, 2011). 

On the other hand, the present study finding showed that khat leaf or its extracts have low overall evidence level in causing oro-pharygeal and oesophageal cancer but moderate to high evidence in killing cancer cells. This finding contradicts our summarized finding that khat is suspected to cause premalignant oral lesions. As a natural product that contains many useful anti-cancer bio-compounds like flavonoids and phenolic compounds (Schmidt-Westhausen et al., 2014; Al-Maweri et al., 2018), it is believed that khat should possess more anti-cancer properties than initiating carcinogenesis. This is supported by the three in vitro studies (Dimba et al., 2004; Bredholt et al., 2009; Lu et al., 2017) which proved that khat alcohol extracts could kill leukemic and breast cancer cells through apoptosis in dose- and time-dependent manner. The only drawback is the no published in vivo study to validate the anti-cancer effects of khat extracts.

Even though the evidences are quite supportive to show that khat is associated with the occurrence of the premalignant oral lesions, but till today, there is no a long-term longitudinal study which could explain the conversion of the khat-induce premalignant oral lesions to malignant oral lesions. Besides, in some of the reported studies that linked khat usage and upper GIT premalignant conditions or cancer (Soufi et al., 1991; Macigo et al., 1995; Ahmed et al., 2010), it was unclear that whether the authors excluded factors like genetics, smoking behaviour and alcohol drinking as other possible reasons which could cause the mentioned conditions and cancer. It has been widely reported that khat consumption is associated with smoking behaviour (Alem et al., 1999; Kebede, 2002; Ayana and Mekonen, 2004; Gorsky et al., 2004; Al-Sanosy, 2009; Douglas et al., 2011; Alsanosy et al., 2013; Kassim et al., 2014; Kubas and Wadi, 2015; Nakajima et al., 2016) and alcohol drinking (Douglas et al., 2011; Reda et al., 2012; Kassa et al., 2016). These two factors had been widely recognized as the risk factors for upper GIT cancer (Morse et al., 2007; Ram et al., 2011; Perry et al., 2015) and if these two factors were not carefully considered in the mentioned studies, this could raise a big bias query. Furthermore, the studies (Kassie et al., 2001; Lukandu et al., 2008) that suggested khat could cause genotoxic effects and oral cancer were in vitro studies and whether or not khat could induce genotoxicity in vivo is still unclear (Widler et al., 1994; Denayer et al., 2014). 

Another point which most authors failed to consider is the widely use of pesticides in khat plantation which had been widely reported (Daba et al., 2011; Ligani and Hussen, 2014; Hassan et al., 2016) and one possible explanation for this phenomenon is the weak legislation and regulation (Daba et al., 2011). Khat chewers who consumed khat grown with pesticides were reported to experience more health complaints on the digestive and respiratory system (Date et al., 2004). Pesticides residues on the khat leaf could be another reason to explain the occurrence of the premalignant oral lesions and upper GIT cancer since pesticides have great potential in inducing genotoxicity (Hassan et al., 2016) and subsequently drives carcinogenesis. Therefore, it is still not 100% to firmly conclude that the reported premalignant oral lesions and cancer were solely caused by khat usage or pesticides. 

Summarizing all the currently available evidences on khat and premalignant oral lesions or cancer, it is important to note that no double-blinded, cohort study has been performed to establish the relationships between khat and cancer. In addition, all reported studies were either case-control or cross-sectional studies (human) or in vitro (cell lines) studies and no animal study has been reported by far. It is therefore recommended that apart from excluding factors which could possibly cause cancer like genetics, smoking, binge drinking and pesticide use in khat plantation, it is also important for the researchers to try animal study or cohort study in further evaluating the relationships between khat and cancer. Besides, longitudinal study should be carried to determine whether khat-induced oral lesion could possibly develop to malignant oral lesion or not in the near future. In addition, before concluding khat is pro- or anti-cancer, it is encouraged that more molecular study could be conducted in the future to show the pathway on how khat causes cancer or eliminates the cancer cells.

In conclusion, RoB and OHAT approaches are methods that could systematically summarize findings from different study findings and this review has employed khat as a study model to determine its relationships with premalignant and cancerous conditions by incorporating RoB and OHAT approaches. From different published findings, khat is said to cause premalignant oral lesions but it is unsure whether these lesions could develop to malignant oral lesions. The currently available evidences are not strong to suggest that khat directly initiates carcinogenesis but some in vitro studies have showed that khat possess some anti-cancer effects instead. In conclusion, the relationship between khat, premalignant conditions and cancer is still not really clear. More study is urged to be conducted in the future to fill in the gap in the literature and to further solve the mystery on whether khat is pro- or anti-cancer. Besides, since the use of pesticides in khat plantation is common, the presence of pesticide residuals in the khat should be investigated through representative sampling of all khat types available in the market, followed by the in-depth study of such residuals to confirm whether these residuals could cause cancer. 
